# Supplementary material for: Mapping the evolution of fertility support policies in China: A content and instrumental analysis
Source: PLoS One. 2025 Oct 9;20(10):e0332137. doi: 10.1371/journal.pone.0332137 (PMC12510515; doi:10.1371/journal.pone.0332137)
Supplement: S1 Appendix — (ZIP) [file pone.0332137.s001.zip › S1 Appendix. 226 original policy documents/11-国家人口和计划生育委员会、教育部、中国扶贫开发协会等关于实施青少年健康人格工程的通知(FBM-CLI-4-139194).doc]

国家人口和计划生育委员会、教育部、中国扶贫开发协会等关于实施青少年健康人格工程的通知

**发布部门：** [国家人口和计划生育委员会(含国家计划生育委员会)(已撤销)](https://www.pkulaw.com/law/chl?Aggs.IssueDepartment=60304) [教育部](https://www.pkulaw.com/law/chl?Aggs.IssueDepartment=60203) [中国扶贫开发协会](https://www.pkulaw.com/law/chl?Aggs.IssueDepartment=705AY) [机构沿革](javascript:void(0))

**发文字号：**人口宣教〔2010〕71号

**发布日期：**2010.09.26

**实施日期：**2010.09.26

**时效性：** [现行有效](https://www.pkulaw.com/law/chl?Aggs.TimelinessDic=01)

**效力级别：** [部门工作文件](https://www.pkulaw.com/law/chl?Aggs.EffectivenessDic=XE0303)

**法规类别：** [人口计划管理](https://www.pkulaw.com/law/chl?Aggs.Category=09701)

国家人口和计划生育委员会 、教育部、中国扶贫开发协会、中国科学技术协会、中国计划生育协会关于实施青少年健康人格工程的通知
（人口宣教〔2010〕71号）

各省、自治区、直辖市人口计生委、教育厅（局）、扶贫开发协会、科学技术协会、计划生育协会：
　　为加强和改进青少年思想道德建设，促使其具备适应现代社会发展的良好心理素质、行为习惯和健康人格，形成有利于青少年健康成长的社会环境，拟在全国逐步实施青少年健康人格工程，并将其纳入提高人口素质、青少年儿童思想品德教育、科学知识普及、家庭文化建设和生育关怀的重要内容。
　　**一、**充分认识实施青少年健康人格工程的重要意义
　　青少年健康人格工程是旨在帮助青少年儿童解除成长中的困惑，培养良好品德情操、心理素质、行为习惯和社会适应能力，提高综合素质的一项社会性文化育人工程。重点包括青少年儿童心理疏导、性健康教育、行为养成、人格塑造和家庭文化建设。实施这项工程，对于加强和改进未成年人思想道德建设，建设青少年的精神家园，深入推进素质教育，帮助青少年学生缓解学习和精神压力，从容应对自身生理和心理变化，适应激烈社会竞争，更快乐、更有效率地学习，更健康地成长，具有重要意义。
　　各级相关部门和机构要从落实科学发展观，构建社会主义和谐社会，推动家庭文明和社会文明，促进人的全面发展，促进人口大国向人力资源强国转变的高度，充分认识实施青少年健康人格工程的重大意义，把它作为一项利国兴邦、造福家庭、惠及后代的基础性工程切实抓紧抓好，抓出成效。

　　**二、**动员全社会广泛参与，稳步实施
　　青少年健康人格工程主要依托社区文化阵地、青少年科技教育基地、人口和计划生育宣传服务机构、计划生育协会工作网络、“青苹果之家”和广大家庭，重点在大、中小学生及其家长中实施。
　　青少年健康人格工程遵循政府主导、社会参与、课外为主、公益运行的原则，在各级党委政府领导下，由相关部门制定规划、政策和运行规则，规范教育内容，建立统一协调的专家咨询服务网络。鼓励支持社区、学校、企业和家庭广泛参与，校园、社区、家庭互动。充分利用社会资源建设“青苹果之家”教育咨询服务机构，在社区文化中心或基层人口和计划生育服务机构建立咨询服务网点。依托人口宣传服务网络、计划生育协会工作网络和青少年活动阵地，借助“家庭人口文化建设”、“家庭互助互学和亲子读书活动”、“青少年假期社会实践”、“主题故事报告会”、“专家名师进校园”及全国咨询服务网，将心理疏导、行为养成、性健康、人格塑造等内容渗透到亿万家庭。
　　青少年健康人格工程首先在条件具备的部分省市试点，取得经验后逐步推广。其目标是：从2010年到2014年，在部分省市及城市社区建立规范的“青苹果之家”咨询服务网络；在各级广播电视台开设讲座或专题节目；制作出版发行科普读物和音像产品；建立为青少年儿童乐于接受的教育方法和模式。在此基础上再用5年时间，借助新媒体和远程教育平台，把教育传播内容普及到农村社区，建立专业化的咨询服务队伍和评估体系。通过实施此项工程，促进广大青少年儿童增强爱国情感，确立远大志向，具备优秀品格，养成良好的心理素质和行为习惯；了解性健康知识，从容面对青春期的生理心理问题；大幅度降低心理疾病发生率和未成年人犯罪率，减少因不科学的性意识和性行为带来的身心伤害，提高综合素质。

　　**三、**加强组织协调，力求实效
　　由国家人口计生委牵头，成立由教育部、中国扶贫开发协会、中国科学技术协会、中国计划生育协会等部门参与的协调小组，负责青少年健康人格工程的组织协调。试点地区也要成立相应的协调机构，开展调查研究，制定规划、政策和措施，协调各方资源，确保在党委政府领导下规范、有序、有效实施。
　　各级人口计生部门要把实施青少年健康人格工程作为统筹解决人口问题，提升人口素质，增强家庭发展能力的一个重要抓手，会同相关部门积极推进青少年儿童心理疏导、行为养成、性健康教育、人格塑造和家庭人口文化建设，由此带动国民整体素质的提高。以独生子女、留守儿童和家长为重点，有针对性地加强教育引导和咨询服务。把实施此项工程与出生缺陷干预、对育龄人群的优质服务、生育关怀、婴幼儿早期教育、新型家庭人口文化建设等紧密结合，完善生命全周期服务模式。利用人口计生系统服务网络优势，依托各级宣传服务机构、人口学校、“青苹果之家”和协会志愿者队伍为青少年儿童提供教育与咨询服务，在全社会倡导优生优育和健康人格。
　　教育、扶贫开发协会、科协、计生协等部门和社会团体要密切配合，发挥各自优势。教育部门要将心理疏导、性健康、行为养成和人格塑造纳入大、中小学德育的重要内容，加强心理咨询室建设和师资培养，指导推动校园课外活动。扶贫开发协会和计生协要动员广大妇女特别是母亲积极参与，不断深化家庭教育，维护青少年儿童在心理、性和生殖健康等方面的合法权益。科协要充分发挥科学家和科普工作者的作用，建立统一的专家咨询库，利用科学技术普及阵地和网络开展对青少年儿童的知识普及和咨询服务。
　　充分利用广播、电视、新闻报刊和门户网站等媒体，加强青少年儿童思想道德和健康人格教育，加强正面引导，扩大公益宣传，开设专题或栏目普及心理、性健康和健康人格的知识，推出适合青少年儿童健康成长的科普影视作品。
　　要保障经费投入，设立专项资金，用于青少年健康人格工程政府倡导的项目。多渠道、多形式开展教育咨询服务，推动公益性文化育人项目的开展。
　　切实加强对青少年健康人格工程的运行指导与监督评估，建立科学的检查评估机制，及时总结经验，发现问题，改进工作，完善管理和服务。
　　联 系 人：中国人口宣传教育中心 马琳
　　联系电话：010-64413274 13801096767

国家人口和计划生育委员会
教育部
中国扶贫开发协会
中国科学技术协会
中国计划生育协会
二○一○年九月二十六日

© 北大法宝：（[www.pkulaw.com](https://www.pkulaw.com/)) 专业提供法律信息、法学知识和法律软件领域各类解决方案。北大法宝为您提供丰富的参考资料，正式引用法规条文时请与标准文本核对。
欢迎查看所有[产品和服务](https://www.pkulaw.com/helps/2.html)。法宝快讯：[如何快速找到您需要的检索结果？](https://www.pkulaw.com/helps/70.html)[法宝](https://www.pkulaw.com/helps/69.html)V6有何新特色？

扫描二维码阅读原文

**原文链接：**<https://www.pkulaw.com/chl/4f8b428adf3d59ffbdfb.html>
